# Adolescent health and well‐being in sub‐Saharan Africa: Strengthening knowledge base and research capacity through a collaborative multi‐country school‐based study

**DOI:** 10.1111/mcn.13411

**Published:** 2023-03-31

**Authors:** Sachin Shinde, Ramadhani Abdallah Noor, Mary Mwanyika‐Sando, Mosa Moshabela, Amare W. Tadesse, Huda Sherfi, Alain Vandormael, Tara Young, Amani Tinkasimile, Roisin Drysdale, Till Baernighausen, Deepika Sharma, Wafaie W. Fawzi

**Affiliations:** ^1^ Department of Global Health and Population, T. H. Chan School of Public Health Harvard University Boston Massachusetts USA; ^2^ Center for Inquiry into Mental Health Pune Maharashtra India; ^3^ United Nations Children's Funds Tanzania; ^4^ Africa Academy of Public Health Dar es Salaam Tanzania; ^5^ College of Health Sciences University of Kwazulu‐Natal South Africa; ^6^ Department of Infectious Disease Epidemiology London School of Hygiene and Tropical Medicine UK; ^7^ Addis Continental Institute of Public Health Addis Ababa Ethiopia; ^8^ School of Health Sciences Ahfad University for Women Sudan; ^9^ Heidelberg Institute of Global Health Heidelberg University Germany; ^10^ DSI‐NRF Centre of Excellence in Human Development University of the Witwatersrand South Africa; ^11^ Africa Health Research Institute South Africa; ^12^ United Nations Children's Funds New York USA; ^13^ Department of Epidemiology, T. H. Chan School of Public Health Harvard University Boston Massachusetts USA; ^14^ Department of Nutrition, T. H. Chan School of Public Health Harvard University Boston Massachusetts USA

**Keywords:** adolescents, cross‐sectional study, health and well‐being, school food environment, sub‐Saharan Africa

## Abstract

In Sub‐Saharan Africa (SSA), adolescents make up around one‐quarter of the population who are growing up in a rapidly urbanizing environment, with its associated risks and benefits, including impacts on health, psychosocial development, nutrition, and education. However, research on adolescents' health and well‐being in SSA is limited. The ARISE (African Research, Implementation Science and Education) Network's Adolescent Health and Nutrition Study is an exploratory, school‐based study of 4988 urban adolescents from five countries: Burkina Faso, Ethiopia, South Africa, Sudan, and Tanzania. A multistage random sampling strategy was used to select the schools and adolescents. Adolescent boys and girls aged 10–15 years were interviewed using a standardized questionnaire by trained enumerators. The questionnaire covered multiple domains including demographic and socioeconomic characteristics, water, sanitation and hygiene practices, antimicrobial resistance, physical activity, dietary behaviours, socioemotional development, educational outcomes, media use, mental health, and menstrual hygiene (only for girls). Additionally, a desk review of health and school meal policies and programs and a qualitative investigation into health and food environments in schools were conducted with students, administrators, and food vendors. In this paper, we describe the study design and questionnaire, present profiles of young adolescents who participated in the study, and share field experiences and lessons learned for future studies. We expect that this study along with other ARISE Network projects will be a first step toward understanding young people's health risks and disease burdens, identifying opportunities for interventions and improving policies, as well as developing potential research capacities on adolescent health and well‐being in the SSA region.

## Key messages

- Several global initiatives recommend greater investment in adolescents' health and well‐being as key to sustainable development.
- Our understanding of how to improve adolescent health and nutrition is limited by a lack of data from low‐ and middle‐income countries.
- The African Research, Implementation Science and Education Network developed a school‐based Adolescent Health and Nutrition Study in five African countries to fill knowledge gaps about young adolescents' health and nutrition.
- Techniques used and the questionnaire of this study may help countries and measurement initiatives focus their efforts and resources on health areas of most potential value for improving health for adolescents.

## 1 INTRODUCTION

Adolescence especially early adolescence (10–14‐year‐old) is a period of rapid physical, emotional, social, and cognitive changes that influence adolescents’ thinking and decision (Ahmed et al., 2015). Understanding these changes and their dynamic extension into young adulthood is an opportunity to reduce health risks and engage young people during formative years that will shape their adulthood and future generations (Haj‐Ahmad & Karmin, 2019; Patton et al., 2016). Therefore, it is crucial to lay the foundation for positive health outcomes and a successful transition to adulthood. As of 2020, there were an estimated 250 million adolescents (aged 10–19 years) in sub‐Saharan Africa (SSA), or 20% of all adolescents worldwide, which is expected to rise to 24% by 2030 (Population Reference Bureau [PRB], 2021; United Nations Department of Economic and Social Affairs Population Division, 2019). Yet, large segments of adolescents in the SSA region are growing up in environments where poverty, unemployment, rapid urbanization, limited educational opportunities, and social instability pose challenges to growth and development (Fox & Gandhi, 2021; UN, 2020; UNICEF, 2021).

Over the past century, women and infants have benefited greatly from improvements in housing, sanitation, maternal education, infant care, vaccination, vector control, and health care systems. However, adolescent health and well‐being remain largely under‐studied (Azzopardi et al., 2019; Mgawadere et al., 2017). In 2016, The Lancet commission on adolescent health and well‐being highlighted several gaps in our understanding of how to improve adolescent health and nutrition (Patton et al., 2016). These included a lack of data from low‐ and middle‐income countries including countries in the SSA region, the need for more adolescent‐friendly interventions, and a global consensus on adolescent research priorities. In its most recent series on adolescent nutrition, the Lancet Commission focused on the role nutrition plays in adolescent growth and development, the role the food environment plays in food choices, and what strategies and interventions might be helpful for healthy adolescent nutrition and growth (Patton et al., 2021).

Yet, the broader context of adolescent health and well‐being in SSA has received little attention, other than research on sexual and reproductive health issues affecting adolescents (Darling, Assefa,  et al., 2020, Darling, Sunguya, et al., 2020; Melesse et al., 2020; United Nations Population Fund and Population Reference Bureau, 2012). A systematic review of existing literature on adolescent sexual and reproductive health in the SSA region indicated only 1302 articles were published between January 2010 and December 2019 (Ajayi et al., 2021). While issues like HIV, sexual behaviours, and access to sexual and reproductive health services received substantial attention, only a few studies focused on early adolescence, large‐scale campaigns, and scaling up of interventions and policy evaluations. Recent studies show that young people in the SSA region are at risk for other factors that adversely affect their health and well‐being, such as nutritional deficiencies, poor eating habits, physical inactivity (Nyundo et al., 2020), poor mental health (Berhane et al., 2020) and injuries (Aboagye et al., 2022), among others. Preventable and treatable, these conditions have an impact on the education sector and are estimated to translate into 200–500 million lost workdays a year (World Bank, 2019). Nevertheless, the health sector in the SSA region lacks adequate investments for school‐going adolescents.

Most SSA countries collect data on adolescents and young people through Demographic and Health Surveys (DHSs), which primarily focus on males and females aged 15–49 (United States Agency for International Development, 2021). In the past 20 years, UNICEF has strategically invested in filling data gaps for monitoring the situation of children and women in developing countries including the SSA region through household‐based Multiple Indicator Cluster Surveys with females and males aged 15–49 (Khan & Hancioglu, 2019). The World Health Organization's Global School‐based student Health Survey (GSHS) is implemented in several countries to describe the prevalence and trends of health‐risk behaviours among adolescents aged 13–17, and to evaluate and improve health‐related policies and programs (World Health Organization, 2019). Consequently, there is a major gap in understanding the health and development of younger adolescents. Although the GSHS surveys are school‐based, they are outdated in some countries and implemented at infrequent intervals in others. Furthermore, none of these surveys addresses school environments and existing policies concerning the health and well‐being indicators of younger adolescents in SSA.

Therefore, the African Research, Implementation Science and Education (ARISE) Network, in partnership with and financial support from UNICEF New York undertook a school‐based Adolescent Health and Nutrition Study in five SSA countries to fill gaps in our understanding of early adolescence, which represents the fastest‐growing youth population of SSA. This paper describes the study design and questionnaire, presents profiles of young adolescents who participated in the study and shares field experiences and lessons learned to inform future research.

## 2 METHODS

The ARISE Network was established in response to a pressing need for a more sustainable and effective public health capacity in Africa (Harvard T. H. Chan School of Public Health, 2014). The Network is led by the Harvard T. H. Chan School of Public Health, United States of America, in partnership with the Africa Academy for Public Health, an independent organization registered in Tanzania, and consists of 21 institutions from nine countries in SSA. Taking advantage of the collaboration between leading African institutions, the ARISE Network enables the region to carry out innovative research and offer cutting‐edge education to develop the next generation of public health leaders in the region.

The ARISE school‐based Adolescent Health and Nutrition Study was conducted in five SSA countries: Ouagadougou, Burkina Faso; Addis Ababa, Ethiopia; Durban, South Africa Khartoum State, Sudan; and Dar es Salaam, Tanzania. We used a mixed‐methods approach to assess the nutrition, health, and school health and nutrition environments of younger adolescents (10–15 years old). We implemented a quantitative survey in each country using a standardized survey questionnaire for younger adolescents. We supplemented the quantitative survey with a qualitative investigation with adolescents, school officials and food vendors. In addition, a desk review of existing national and subnational policies, guidelines, regulations and by‐laws related to adolescent health in each of these countries was conducted.

### 2.1 Sampling

We sampled schools and students at each site by using a multistage cluster random sampling technique. To begin, we randomly selected districts and administrative units in the selected cities of each country. We then randomly selected around 20 primary schools per country. Approximately 60 students per school were randomly selected, resulting in a sample of nearly 1200 adolescents per country. Male and female students aged 10–14 years were invited to participate in Ethiopia, South Africa, Sudan and Tanzania, while in Burkina Faso, male and female students aged 11–15 years were recruited. We excluded those adolescents who refused to participate, were too ill to be interviewed or were absent during data collection. Data collection occurred between March and December 2020.

In total, 4988 adolescents participated in survey interviews in five countries. Our sample of adolescents included 46% boys and 54% girls. The mean age of the participants was 12.2 years (SD of 1.2). Table 1 shows the adolescent survey samples and demographic characteristics of participants enrolled in each country.

**Table 1:** Demographic characteristics of participants enrolled in each country of the ARISE Network School‐based Adolescent Health Study.

City and country	Number of districts/localities per country	Number of schools per district	Total schools	Grades covered	Number of adolescents recruited per school	Total number of adolescents recruited per country	Females *f* (%)	Mean age in years (SD)
Ouagadougou, Burkina Faso	5	4	20	3–6	60	1059	603 (56.9)	13.5 (1.2)
Addis Ababa, Ethiopia	10	2	20	5–8	60	1200	657 (54.8)	12.6 (1.2)
Durban, South Africa	2	10	20	5–7	60	371	209 (56.2)	11.5 (1.2)
Khartoum State, Sudan	4	3	12	5–8	60–100	1101	553 (50.2)	12.1 (1.3)
Dar es Salaam, Tanzania	5	4	20	4–6	60–70	1257	656 (52,2)	11.6 (1.2)

The qualitative component of the study involved face‐to‐face interviews with senior administrators or teachers in selected schools across the countries. Additionally, four focus group discussions were conducted with adolescents in each country, each with six participants. Out of 20 schools selected for the adolescent survey, 4 schools per country were purposefully chosen for focus groups. At each selected school, teachers recommended adolescents for group discussions. Of the 20 schools selected for the adolescent survey, we randomly selected 10 schools for interviews with food vendors in each country. In every selected school, formal and informal food vendors in and around the school were interviewed using a semistructured questionnaire.

### 2.2 Settings and sampling procedures

This study was performed in partnership with the *Institut Supérieur des Sciences de la Population* (Burkina Faso), Addis Continental Institute of Public Health (Ethiopia), Institute for Global Health at the University of Heidelberg (Germany), University of KwaZulu‐Natal (South Africa), Ahfad University for Women (Sudan), and Africa Academy of Public Health (Tanzania). Study sites were selected based on budget allocations, infrastructure, networking with government agencies and schools, research team capacity and willingness to participate. Below are descriptions of each site and its specific procedures.

#### 2.2.1 Burkina Faso

Burkina Faso is located in West Africa, with 50% of its population under 15 years (United Nations Department of Economic and Social Affairs Population Division, 2019). It is ranked 114th on the Human Development Index for 2019 (United Nations Development Programme, 2018). Despite recent efforts to improve its education, the country's adult literacy rate is among the lowest in the world (41%) (UNESCO Institute for Statistics, 2021). The study was conducted in the capital city of Burkina Faso, Ouagadougou, which is divided into five arrondissements: Baskuy, Nongr‐Massom, Boulmiougou, Bogodogo and Sig‐Nonghin. We used a two‐stage sampling process to select secondary schools and adolescents in five arrondissements of Ouagadougou. During the 2018–2019 school year, there were 251 schools with more than 200 adolescents aged 11–15 in the five arrondissements (Ministry of Education Government of Burkina Faso, 2017). In the first stage, 22 secondary schools with grades 3–6 were chosen at random from 251 schools. During the next stage, 1059 adolescents were recruited from 22 schools using proportionate representative sampling. Students' registration details including name, age, sex and grade were used to draw the sample.

#### 2.2.2 Ethiopia

Ethiopia is the second‐most populous country in East Africa, with 114 million people and more than 40% of them under the age of 15 (United Nations Department of Economic and Social Affairs Population Division, 2019). Although the Ethiopian economy has grown by 10% over the past decade, it is ranked 179th on the Human Development Index for 2019 (United Nations Development Programme, 2018). Ethiopia has one of the world's lowest adult literacy rates (52%) (UNESCO Institute for Statistics, 2021). During the 2019–2020 academic year, the quantitative survey of adolescents was conducted in 20 public primary schools in Addis Ababa, Ethiopia's capital city. Ten boroughs make up the city. First, we randomly selected 2 schools from each of Addis Ababa's 10 boroughs. We then randomly selected 60 adolescent boys and girls from grades 5 to 8 in each school (15 students per grade). Thus, we surveyed 1200 adolescents in Ethiopia. Students' registration details, including name, age and sex, were used as a sampling frame.

#### 2.2.3 South Africa

South Africa, the southernmost point of Africa, has 59 million residents, 21% of whom are adolescents (United Nations Department of Economic and Social Affairs Population Division, 2019). The country's GDP per capita is relatively high compared to other countries in SSA, and it is ranked 114th on the Human Development Index for 2019 (United Nations Development Programme, 2018). South Africa has a literacy rate of 87% (UNESCO Institute for Statistics, 2021). The study was conducted in the two educational districts of the EThekwini metropolitan municipality of the KwaZulu‐Natal province, namely Pinetown and Umlazi. First, we selected 10 schools from each district at random. Next, a total of 60 students were randomly selected from grades 5 to 7 (20 students per grade) using student class lists. A total of 371 students were interviewed in March 2020. Data collection could not be completed because schools were closed due to the COVID‐19 outbreak.

#### 2.2.4 Sudan

Sudan is located in northeast Africa, with more than 20% of its population between ages 10 and 19 years (United Nations Department of Economic and Social Affairs Population Division, 2019). Sudan ranks 167th out of 189 countries in the 2019 Human Development Index (United Nations Development Programme, 2018). By 2018, Sudan had a literacy rate of 61% (UNESCO Institute for Statistics, 2021). The ARISE adolescent survey was conducted in Khartoum State during the 2019–2020 academic year. According to administrative divisions, the state is divided into seven localities, four of which were selected for the adolescent survey. They were Bahri, Omdurman, Karrari and Umbada. Next, three public primary schools were randomly selected in each locality. At each school, 60–100 adolescent boys and girls were randomly selected from grades 5 to 8. A class roster was used to recruit 15–25 students per grade, yielding 1100 adolescents in Khartoum State, Sudan.

#### 2.2.5 Tanzania

Tanzania has a population of 54 million and 12 million of these are adolescents (United Nations Department of Economic and Social Affairs Population Division, 2019). Among 189 countries, Tanzania placed 163rd on the Human Development Index in 2019 (United Nations Development Programme, 2018). Tanzania's literacy rate for 2020 is 78% (UNESCO Institute for Statistics, 2021). The cross‐sectional survey of adolescents was conducted in Dar es Salaam, which is divided into five administrative districts: Kinondoni, Ilala, Temeke, Ubungo and Kigamboni. A total of 20 public primary schools, 4 from each district, were chosen at random. Within each school, around 60 boys and girls were randomly selected from grades 4 to 6, yielding a total sample of 1257.

### 2.3 Ethical considerations

We engaged and consulted appropriate authorities in each country during the design phase and prior field activities. Approvals were obtained from the relevant national and local ministries or authorities, institutional review boards and participating schools. Supporting Information: Appendix 1 provides more information about institutional approvals. All participants and their parents or guardians were informed of the purpose and nature of the study and voluntary participation. Written parental consent and adolescent assent were obtained from all adolescent participants. Written consent was also obtained from school officials and food vendors.

### 2.4 Questionnaire

#### 2.4.1 Description of the adolescent survey questionnaire

Adolescents were interviewed face‐to‐face by trained interviewers using a standardized questionnaire translated into the local language, namely French in Burkina Faso, Amharic in Ethiopia, isiZulu in South Africa, Arabic in Sudan and Swahili in Tanzania. The questionnaire was adapted from the GSHS questionnaire (World Health Organization, 2019), and the ARISE Network community‐based Adolescent Health Study questionnaire (Darling, Assefa, et al., 2020). The GSHS questionnaire has been validated and applied in 22 countries across SSA, including Ethiopia, South Africa, Sudan and Tanzania. Burkina Faso is the only country in the current study that did not participate in the GSHS. The community‐based ARISE Adolescent Health Study questionnaire was administered in nine countries in SSA, including Ethiopia, Burkina Faso and Tanzania.

Additionally, our study team reviewed the content of the guidelines for assessing nutrition‐related knowledge, attitudes and practices by the Food and Agriculture Organization of the United Nations (Kennedy et al., 2010), Household Hunger Scale of Food and Nutrition Technical Assistance, United States Agency for International Development (Ballard et al., 2011), Global Early Adolescent Study toolkit (Chandra‐Mouli et al., 2017) and the learning resources of the Southern and Eastern Africa Consortium for Monitoring Educational Quality (Southern and Eastern Africa Consortium for Monitoring Educational Quality, 2019) to develop and ensure an appropriate survey questionnaire for 10–14 years old.

Modules covered by the questionnaire included demographics, socio‐economy, water, sanitation and hygiene (WASH) practices, antimicrobial resistance, physical activity, diet and nutrition, socio‐emotional development, educational outcomes and learning resources, media use, and mental health, and menstrual hygiene (only to girls). The sites included various insertions to capture the types of food and activity specific to their sites, as well as specific slang words and terminology used to describe certain issues. Table 2 provides a detailed description of each module covered in the questionnaire, and the questionnaire is attached as Supporting Information: Appendix 2.

**Table 2:** A summary of modules included in the ARISE Network School‐based Adolescent Health Study questionnaire

Module	Subtopic	Items	Source
Demographics		Age Sex Current grade Involvement in paid work Parental vital status Parental education and occupation Number of siblings Time to reach school Transportation to school School arrival and leaving time	ARISE Network Adolescent Health Study Questionnaire Global School‐based Student Health Survey
Socio‐economy		Cooking fuel use Frequency of household annual vacation Household assets	ARISE Network Adolescent Health Study Questionnaire
Water, sanitation and hygiene (WASH)	Household drinking water supply	Source of drinking water Drinking water treatment practice	ARISE Network Adolescent Health Study Questionnaire
	Household sanitation facility	Type of toilet facility Sharing of toilet facility	
	Dental hygiene	Frequency of tooth brushing Frequency of dental visits	
	Hand hygiene	Frequency of handwashing before eating Frequency of handwashing after using the toilet Use of soap for handwashing	
Antimicrobial resistance	Knowledge and use of antibiotics	Knowledge of antibiotics Ever used antibiotics Last use of antibiotics Reasons for last use of antibiotics	ARISE Network Adolescent Health Study Questionnaire
Reproductive health (Only to girls)	Menstruation	Knowledge of menstruation First learning source Menarche status Menstrual hygiene practices Menstruation‐related school absence	ARISE Network Adolescent Health Study Questionnaire
Physical activity		Frequency of physical activity Frequency of walking/biking to and fro school Frequency of participation in a physical education class	ARISE Network Adolescent Health Study Questionnaire
Diet and nutrition	Eating behaviours	Food group consumption in the last 24 h Timing and location of food consumption	FAO Household and Individual Dietary Diversity Questionnaire
	Dietary quality	Frequency of healthy and unhealthy foods consumption	Global Diet Quality Score Questionnaire
	Food security	Frequency of household food insecurity	USAID FANTA Household Hunger Scale
Socio‐emotional development	Socio‐emotional development	Internalizing problems (emotional and peer problems) Externalizing problems (hyperactivity and conduct problems) Pro‐social behaviour	Strengths and Difficulties Questionnaire
	Violence	Ever involved in a physical fight Frequency of involvement in physical fights Number of male and female friends	ARISE Network Adolescent Health Study Questionnaire
Educational outcomes and learning resources	School connectedness and attendance	Availability of caring adults in school Frequency of school absence Reasons for school absence	Global Early Adolescent Study
	Academic outcome	Frequency of grade repetition	Southern and Eastern Africa Consortium for Monitoring Educational Quality
	Learning resources	Number of books at home	
Media	Access to and use of media	Access to a computer, laptop, or tablet Own cell or mobile phone Access to other's cell or mobile Access to social media	Global Early Adolescent Study
Mental health	Depression	Symptoms of depression	Patient Health Questionnaire
Anthropometric measures and hemoglobin		Height Weight Hemoglobin level in blood	‐

Demographic information collected included the age and gender of participants, current grade enrollment, engagement in paid work, people currently living with, parental education and occupation, number of siblings, transportation to reach school, and arrival and departure time to school. The socio‐economy module includes questions on the type of fuel used for cooking, annual vacation, and assets owned by the household.

The module on WASH included questions on the source of drinking water for the household, actions taken to make the water safe to drink by the household, toilet facility, dental hygiene, and handwashing habits. The module on antimicrobial resistance comprised questions on awareness and usage of antibiotics including the usage and reasons for usage.

The module on female menstruation included questions on awareness of menstruation, the onset of menstruation, hygiene practices during menstruation and missing school due to menstruation. The module on physical activity asked about the number of days participant was physically active in the past week, the number of days walked or cycled in the past week and engagement in physical education class.

The module on dietary behaviours included questions on the consumption of food groups, the quality of food, and the timing and location of food consumption in the last 24 h. The Global Diet Quality Score questionnaire was used to assess the diet quality based on the consumption of healthy and unhealthy foods over the last 7 days (Bromage et al., 2021). The questionnaire classified foods as healthy and unhealthy based on two major considerations: (1) data from the literature on the direction of association with the risk of noncommunicable diseases and (2) nutrient contribution in the worldwide setting. The healthy food groups include (1) dark leafy green vegetables, (2) cruciferous vegetables, (3) deep orange vegetables, (4) deep orange fruits, (5) deep orange tubers, (6) other vegetables, (7) citrus fruits, (8) other fruits, (9) legumes, (10) nuts and seeds, (11) poultry, (12) fish, (13) whole grains, (14) liquid oils, (15) low‐fat dairy and (16) eggs. The unhealthy food groups include (1) white roots and tubers, (2) red meat, (3) processed meat, (4) refined grains and baked goods, (5) sugar‐sweetened beverages, (6) sweets and ice cream, (7) high‐fat dairy and (8) juices. The fried food eaten away from home is included as a double‐counted component. The food groups are then scored based on healthy and unhealthy food categories (e.g., never, once/week, 2–4 times/week, 5–7 times/week or once/day). The total score ranges from 0 to 49, with a higher score indicating a healthier diet. The dietary behaviours module also contains questions on household food security in the past month.

The module on socio‐emotional development includes the adolescent version of the Strengths and Difficulties Questionnaire (SDQ) to assess the negative and positive behavioural attributes (Goodman, 2001). The recall period for the questionnaire is last 6 months. Each item in the questionnaire has a response option of ‘not true’, ‘somewhat true’ or ‘definitely true’. The 25 items in the SDQ are divided into five scales of five items each, including hyperactivity, conduct problems, emotional problems, peer problems and prosocial behaviour. For each of the five scales, the total score ranges from 0 to 10 if all five items are answered. Summing the scores on all scales except the prosocial scale yields a total difficulty score. The resulting difficulty scores range from 0 to 40, with higher scores indicating more difficulty. Although not validated, the SDQ is one of the most widely used measures of psychopathology among children and adolescents globally and in SSA. It has been translated into several languages, including those spoken in study settings (e.g., Amharic in Ethiopia, Zulu in South Africa and French in Burkina Faso) (Goodman, 2001; Youth in Mind, 2022). This module also includes questions on engagement in physical fights and information on friends.

The module on educational outcomes and learning resources contains questions on the availability of caring adult in school, frequency of and reasons for school absence, frequency of grade repetition and availability of learning resources at home. The module on media includes questions on access to digital technology including a computer, a laptop, mobile phones and social media platforms.

The module on mental health asks questions measuring depressive symptoms using the 6‐item version of the Patient Health Questionnaire (Kroenke et al., 2001). The recall period for the questionnaire is the last 2 weeks and the response set for the items is ‘not at all’, ‘several days’, ‘more than half of the days’ and ‘nearly every day’. The total score ranges from 0 to 18 with higher scores indicating more severe symptoms. The Patient Health Questionnaire is widely used to measure depression among adolescents across the globe, but the abbreviated and complete versions have not been validated in the study setting.

The adolescent survey also included haemoglobin and anthropometric measurements. Trained interviewers measured the height and weight of all the adolescent participants using digital scales and stadiometers. Each anthropometric measurement was repeated twice per participant to ensure accuracy. The interviewers also collected adolescents' blood samples to measure haemoglobin levels to estimate the prevalence of anaemia. A portable HemoCue instrument, with capillary samples inserted into a microcuvette, was used to measure haemoglobin levels on the spot.

The study team comprises experts from across health and nutrition domains, including adolescent development and health, diet and nutrition, the food environment, mental health, reproductive health, and adolescent health policies and programs. These team members provided valuable insights into school contexts and adolescence in selected countries. We discussed the draft tool in detail at a joint meeting in April 2019. The team recommended revisions to the draft questions and the addition of validated instruments or questions where needed. All members of the research team reviewed the revised draft instrument by consensus before final approval.

At each location, interviewers were trained for 3 days with lectures and interactive sessions. We attempted to help interviewers overcome their reluctance to discuss sensitive topics and provided training on what to do if a participant was distressed or uncomfortable. The interviewers were familiarized with each module of the questionnaire, concepts and questions, and their rationale. We conducted role‐plays and mock interviews based on each module. During the interviewer training for collecting biomarkers and anthropometrics, the methods of blood collection, haemoglobin measurement, height, and weight measurements, calibration of weighing scales and stadiometers, ethical requirements, and biohazard waste disposal were covered.

#### 2.4.2 Description of the qualitative investigation

The qualitative investigation was aimed at understanding the school health and nutrition policies and guidelines and school environment in terms of the food environment, physical activity and WASH practices for adolescents. Group discussion with adolescents covered the following topics: health literacy, influences of school on health and nutrition, eating habits and preferences, peer pressure, and peer influences on health and nutrition. School administrators and teachers were interviewed to understand school health and nutrition policies and guidelines, as well as WASH practices. Food vendors were interviewed to learn about the availability of different foods and beverages around school premises.

We also reviewed national and subnational policies related to school meals and food environments in five countries. Documents related to adolescent health and school health programs were searched, including national/regional policies, interventions, guidelines, regulations and by‐laws. Google search served as a starting point in searching documents, which was supplemented by contacts with specialized ministries and institutions at the national level in each country, as well as searches on the institutional databases of the World Health Organization, United Nations Children's Fund, United Nations Educational, Scientific and Cultural Organization, United Nations Fund for Population Activities and World Bank. This review focused on understanding the availability of school meal programs, nutritional standards of the meals offered in schools, training of service staff in healthy food preparation and food safety, financial support for school meal programs, availability of clean and safe drinking water in the schools, availability of healthy and unhealthy foods in the school cafeterias as well as with surrounding vendors and information on other relevant interventions such as school garden and nutrition education.

### 2.5 Data management

The Open Data Kit program (ODK version 1.2.2) and tablet‐assisted personal interviewing technique were used to collect quantitative data. This method eliminates the need to enter data on paper during interviews and includes in‐built quality checks. Qualitative interviews and group discussions were recorded and transcribed verbatim. Identifiable data was stored in secure databases accessible only to the study team. Interviewers and supervisors were trained in research ethics, consenting procedures, and the collection of health data, including dietary assessments, anthropometric measurements and point‐of‐care hemoglobin assessments. Each ARISE local partner monitored data collection both internally and at ARISE Network meetings. The data were combined and cleaned centrally using STATA 14 (Version 14).

## 3 DISCUSSION

The growing population of youth in the SSA region necessitates drastic measures to ensure that their health, diet and nutrition, education, employment and social needs are met (United Nations Department of Economic and Social Affairs Population Division, 2015). Improving the health of adolescents in the region is critical if the world is to achieve the United Nations Sustainable Development Goals and the specific targets and goals mentioned in the Secretary General's Global Strategy for Women's, Children's and Adolescents' Health (Kuruvilla et al., 2016). With an approximate 91% school enrollment rate across the world, the Lancet Commission of Adolescent Health recommends integrating health and education interventions at the school to meet the needs of adolescents, and to create an environment that facilitates the demographic dividend effect (Patton et al., 2016, 2021). However, despite an increased call for research on the health and well‐being of adolescents and young people to guide global and national initiatives, there is limited research on adolescent health and nutrition in the SSA region (World Bank, 2019).

The ARISE Network serves as a platform for a wide range of research and cutting‐edge education in key priority areas including adolescent health and nutrition in the SSA region. With community‐ (Darling, Assefa, et al., 2020) and school‐based adolescent health surveys, the Network is filling a critical knowledge gap by identifying the specific needs of out‐of‐school and school‐going adolescents. The Network is exploring the effects of various risk factors on health behaviours and outcomes including diet, nutrition, physical activity, non‐communicable diseases, mental health, substance abuse, sexual behaviour and reproductive health, and health care access and utilization. The region lacks strong research programs that bring together disciplines that are important for a comprehensive understanding of the drivers of young people's health, as opposed to other regions of the world that have several centres of excellence focused on young people's health. This gap is being bridged by the Network through strengthening multidisciplinary collaborations on adolescent health in African universities and increasing collaborations between institutions that have extensive research experience in this area. To the best of our knowledge, this is the first large school‐based survey on younger adolescents' health and nutrition in the region that includes policy and school environment assessment.

While the Global Strategy for Women's, Children's, and Adolescents' Health recognized the need for sound health data to promote accountability (Kuruvilla et al., 2016), there are few standardized tools available to assess health, nutrition and well‐being of adolescents in SSA. In this effort, we developed and pretested tools specific to SSA, incorporating input and reviews from each participant country. We expect that the resulting tools of our previous and current efforts will be integrated with other evidence generation efforts including the National School Health and Nutrition Surveys and the DHS Surveys, thereby improving the generation of evidence required to develop nutrition and health programs for adolescents throughout the region.

Although the school‐based surveys provide valuable health and education data on adolescents to inform future interventions, they pose several challenges. Our experience revealed challenges that were both about ethics and systemic problems. From an ethical standpoint, it is particularly important to take care when interviewing adolescents in school settings about sensitive topics such as mental health and reproductive health. Particularly important to response validity is protection from intrusion by fellow students, teachers or other staff (Eder & Fingerson, 2001). In addition, some participants may feel uncomfortable being asked sensitive questions. Given this possibility, we matched interviewers to participants based on sex. Parents were informed during the consent process to ensure that the adolescents were protected from harm. As part of the consent process, the adolescents were also informed of the purpose and nature of the study and their voluntary involvement was stressed and respected.

In studies such as ours that rely on self‐reported attitudes and behaviours, social desirability bias can be problematic (Richman et al., 1999). Respondents could purposefully misreport stigmatized behaviours or report normative ones under pretences, if the actual behaviour would be viewed as socially unacceptable, particularly in mental health and eating habits questions. This may worsen in survey questionnaires administered by interviewers since the participant may be reluctant to answer sensitive questions out of fear of judgement on the interviewer's part. We attempted to reduce social desirability bias by assuring absolute anonymity. Participants were informed that their responses would not be linked to their personal information, kept completely confidential, and would not be shared with anyone except members of the research team.

Any school‐based research can be extremely challenging to schedule. We consulted school staff and district calendars to plan around dates like examination days, holidays, breaks, teacher training, and sporadic events. Despite our best efforts, we still faced unexpected schedule‐related challenges. Some of these challenges were caused by events known to school staff but not taken into account when setting the data collection dates, and others were caused by unexpected events such as a lockdown caused by COVID‐19. Therefore, we have developed a checklist (Supporting Information: Appendix 3) to elicit not only general information about school schedules that may prevent us from reaching students during the designated dates and times but also information regarding specific activities such as designated days, periods, planned and unplanned events and field trips.

We planned to survey randomly selected students from a few classes. Despite receiving rosters with all students' classroom assignments, we found it difficult to identify and separate groups of students seated in classrooms or engaged in other activities for the survey interviews. Another challenge was ensuring the highest quality of data was collected with as little disruption to the school schedule as possible. Disruptions may be the result of multiple factors, such as large school campuses complicating the logistics arrangements, mixed readiness of students and teachers for disruptions, and a lack of privacy and physical space in the classroom. We aimed to minimize disruptions at the school by going in and out quickly. It was useful to tour the campus in advance and decide where to position the research team at the classroom level to reduce time in the school for the preparation and administration of the survey. It was equally important to establish a nodal contact point at each school to address both space‐related and time‐related concerns efficiently.

This study has a few limitations. First, we sampled school‐going adolescents aged 10–15, which may not be representative of the general population in early adolescence within the five countries or the SSA region overall. We nevertheless believe that the thoughtful study design, careful collection of data, and appropriate statistical analysis improve the internal validity of our study and the possibility that the results may apply to adolescents from different backgrounds. Furthermore, cross‐country comparisons can provide meaningful inferences that may be applied to other adolescent populations in the region with similar demographic and geographical characteristics.

Second, it is important to consider the limitations of cross‐sectional data. The temporal relationship between predictors and health and nutrition outcomes cannot be determined with certainty. However, studies on the health and nutrition domains selected for this study are of importance in public health for assessing the burden of disease in the adolescent population and for planning and allocating health resources. Moreover, the data collected through this study can be used to plan and deliver health and education services to both school‐going and out‐of‐school youth.

Third, this study collected mainly quantitative data on adolescents. Although we used a limited number of small group discussions with adolescents, they mostly focused on exploring food habits and the school food environment. There may have been valuable information gained if qualitative data collection had been done with multiple stakeholders, including parents, educational communities, community representatives, health care workers, and policy and program makers and implementers; however, it was neither feasible nor preferable at this exploratory stage. Nevertheless, we were able to standardize data collection across five different sites by using a structured questionnaire. These data can also be used in future surveys as baseline data to track progress toward the health and nutrition outcomes of adolescents.

Lastly, the instrument was not validated; however, the instrument was based on one used in the ARISE Network's community‐based survey among adolescents aged 10–19 in nine communities in seven SSA countries, and other validated and/or widely used instruments around the world.

### 3.1 Future directions

In collaboration with partner organizations, ARISE Network is currently designing and evaluating demonstration projects in schools and communities that aim to improve adolescent health and nutrition in the SSA region. It is hoped that the tools developed will be integrated into existing population‐based surveys like Health and Demographic Surveillance Systems and schools, which will enable low‐cost monitoring of trends in adolescents' health and nutrition. Collectively, the evidence generated from this effort and future efforts will inform national programs on incorporating interventions for adolescents. Additionally, we will extend our reach to several countries in the region and use innovative and digital methods in the future to reduce reporting bias and social desirability bias. Furthermore, we will strengthen the region's capacity for generating rigorous scientific evidence and develop innovative ways to encourage the utilization of this evidence by national authorities, policymakers, and key stakeholders to inform evidence‐based policies and programs designed to improve the health and well‐being of young people. To help us capitalize on the dividends of a growing young population, the ARISE Network will continue to work with government and nongovernment agencies, as well as funding agencies in SSA.

## AUTHOR CONTRIBUTIONS

Wafaie W. Fawzi, Ramadhani Abdallah Noor, Mary Mwanyika‐Sando, Mosa Moshabela, Amare Worku Tadesse, Huda Sherfi, Till Baernighausen, Alain Vandormae, Roisin Drysdale, and Deepika Sharma designed the study and related material. Mary Mwanyika‐Sando, Amare Worku Tadesse,  Huda Sherfi, Alain Vandormae, Roisin Drysdale, Tara Young, and Amani Tinkasimile performed the research. Sachin Shinde and Ramadhani Abdallah Noor wrote the first draft of the manuscript. All authors reviewed and critically revised the manuscript, and have read and approved the final manuscript.

## CONFLICT OF INTEREST

The authors declare no conflict of interest.

## Supporting information

Supplementary information.

## Data Availability

Data described in the manuscript, code book, and analytic code will be made available upon request pending application and approval by the study team.

## References

[mcn13411-bib-0001] Aboagye, R. G. , Mireku, D. O. , Nsiah, J. J. , Ahinkorah, B. O. , Frimpong, J. B. , Hagan, J. E., Jr. , Abodey, E. , & Seidu, A. (2022). Prevalence and psychosocial factors associated with serious injuries among in‐school adolescents in eight sub‐Saharan African countries. BMC Public Health, 22, 853. 10.1186/s12889-022-13198-6 35484506 PMC9047327

[mcn13411-bib-0002] Ahmed, S. P. , Bittencourt‐Hewitt, A. , & Sebastian, C. L. (2015). Neurocognitive bases of emotion regulation development in adolescence. Developmental Cognitive Neuroscience, 15, 11–25. 10.1016/j.dcn.2015.07.006 26340451 PMC6989808

[mcn13411-bib-0003] Ajayi, A. I. , Otukpa, E. O. , Mwoka, M. , Kabiru, C. W. , & Ushie, B. A. (2021). Adolescent sexual and reproductive health research in sub‐Saharan Africa: A scoping review of substantive focus, research volume, geographic distribution, and Africa‐led inquiry. BMJ Global Health, 6, e004129. 10.1136/bmjgh-2020-004129 PMC7878134 33568395

[mcn13411-bib-0004] Azzopardi, P. S. , Hearps, S. , Francis, K. L. , Kennedy, E. C. , Mokdad, A. H. , Kassebaum, N. J. , Lim, S. , Irvine, C. , Vos, T. , Brown, A. D. , Dogra, S. , Kinner, S. A. , Kaoma, N. S. , Naguib, M. , Reavley, N. J. , Requejo, J. , Santelli, J. S. , Sawyer, S. M. , Skirbekk, V. , … Patton, G. C. (2019). Progress in adolescent health and wellbeing: Tracking 12 headline indicators for 195 countries and territories, 1990‐2016. Lancet, 393(1076), 1101–1118. 30876706 10.1016/S0140-6736(18)32427-9 PMC6429986

[mcn13411-bib-0005] Ballard, T. , Coates, J. , Swindale, A. , & Deitchler, M. (2011). *Household Hunger Scale: Indicator definition and measurement guide*. FHI 360, Food and Nutrition Technical Assistance and USAID.

[mcn13411-bib-0006] Berhane, Y. , Canavan, C. R. , Darling, A. M. , Sudfeld, C. R. , Vuai, S. , Adanu, R. , Bärnighausen, T. , Dessie, Y. , Bukenya, J. N. , Guwatudde, D. , Killewo, J. , Sando, M. M. , Sie, A. , Oduola, A. , & Fawzi, W. W. (2020). The age of opportunity: Prevalence of key risk factors among adolescents 10‐19 years of age in nine communities in sub‐Saharan Africa. Tropical Medicine and International Health, 25(1), 15–32. 10.1111/tmi.13339 31698531

[mcn13411-bib-0007] Bromage, S. , Batis, C. , Bhupathiraju, S. N. , Fawzi, W. W. , Fung, T. T. , Li, Y. , Deitchler, M. , Angulo, E. , Birk, N. , Castellanos‐Gutiérrez, A. , He, Y. , Fang, Y. , Matsuzaki, M. , Zhang, Y. , Moursi, M. , Gicevic, S. , Holmes, M. D. , Isanaka, S. , Kinra, S. , … Willett, W. C. (2021). Development and validation of a novel food‐based Global Diet Quality Score (GDQS. Journal of Nutrition, 151(2), 75S–92S. 10.1093/jn/nxab244 34689200 PMC8542096

[mcn13411-bib-0008] Chandra‐Mouli, V. , Plesons, M. , Adebayo, E. , Amin, A. , Avni, M. , Kraft, J. M. , Lane, C. , Brundage, C. L. , Kreinin, T. , Bosworth, E. , Garcia‐Moreno, C. , & Malarcher, S. (2017). Implications of the Global Early Adolescent Study's formative research findings for action and research. Journal of Adolescent Health, 61(4S), 5–9. 10.1016/j.jadohealth.2017.07.012 PMC5612022 28915994

[mcn13411-bib-0009] Darling, A. M. , Assefa, N. , Bärnighausen, T. , Berhane, Y. , Canavan, C. R. , Guwatudde, D. , & Fawzi, W. W. (2020). Design and field methods of the ARISE Network Adolescent Health Study. Tropical Medicine and International Health, 25(1), 5–14. 10.1111/tmi.13327 31691409

[mcn13411-bib-0010] Darling, A. M. , Sunguya, B. , Ismail, A. , Manu, C. , Canavan, C. , Assefa, N. , & Guwattude, D. (2020). Gender differences in nutritional status, diet and physical activity among adolescents in eight countries in sub‐Saharan Africa. Tropical Medicine and International Health, 25(1), 33–43. 10.1111/tmi.13330 31693777

[mcn13411-bib-0012] Eder, D. , & Fingerson, L. (2001). Interviewing children and adolescents. In J. F. Gubrium & J. A. Holstein (Eds.), Handbook of interview research: Context and method. Sage Publications.

[mcn13411-bib-0013] Fox, L. , & Gandhi, D. (2021). *Youth employment in sub‐Saharan Africa: Progress and prospects*. AGI working paper #28. Africa Growth Initiative, Brookings Institute.

[mcn13411-bib-0014] Goodman, R. (2001). Psychometric properties of the Strengths and Difficulties Questionnaire. Child and Adolescent Psychiatry, 40(1), 1337–1345. 10.1097/00004583-200111000-00015 11699809

[mcn13411-bib-0015] Haj‐Ahmad, J. , & Karmin, S. (2019). Adolescents and a sustainable future: An investment opportunity for the private sector. UNICEF.

[mcn13411-bib-0016] Harvard T. H. Chan School of Public Health . (2014). *Africa Research, Implementation Science and Education (ARISE) Network*. https://www.hsph.harvard.edu/africa-health-partnership/partnerships/

[mcn13411-bib-0017] Kennedy, G. , Ballard, T. , & Dop, M. C. (2010). Guidelines for measuring household and individual dietary diversity. Food and Agriculture Organization.

[mcn13411-bib-0018] Khan, S. , & Hancioglu, A. (2019). Multiple indicator cluster surveys: Delivering robust data on children and women across the globe. Studies in Family Planning, 50(3), 279–286. 10.1111/sifp.12103 31486080 PMC6771654

[mcn13411-bib-0019] Kroenke, K. , Spitzer, R. L. , & Williams, J. B. (2001). The PHQ‐9: Validity of a brief depression severity measure. Journal of General Internal Medicine, 16(9), 606–613. 10.1046/j.1525-1497.2001.016009606.x 11556941 PMC1495268

[mcn13411-bib-0020] Kuruvilla, S. , Bustreo, F. , Kuo, T. , Mishra, C. , Taylor, K. , Fogstad, H. , Gupta, G. R. , Gilmore, K. , Temmerman, M. , Thomas, J. , Rasanathan, K. , Chaiban, T. , Mohan, A. , Gruending, A. , Schweitzer, J. , Dini, H. S. , Borrazzo, J. , Fassil, H. , Gronseth, L. , … Costello, A. (2016). The global strategy for women's children's and adolescents' health: A roadmap based on evidence and country experience. Bulletin of World Health Organization, 94, 398–400. 10.2471/BLT.16.170431 PMC4850541 27147772

[mcn13411-bib-0021] Melesse, D. Y. , Mutua, M. K. , Choudhury, A. , Wado, Y. D. , Faye, C. M. , Neal, S. , & Boerma, T. (2020). Adolescent sexual and reproductive health in sub‐Saharan Africa: Who is left behind? BMJ Global Health, 5, e002231. 10.1136/bmjgh-2019-002231 PMC7042602 32133182

[mcn13411-bib-0022] Mgawadere, F. , Kana, T. , & van dec Broek, N. (2017). Measuring maternal mortality: A systematic review of methods used to obtain estimates of the maternal mortality ratio (MMR) in low‐ and middle‐income countries. British Medical Bulletin, 121(1), 121–134. 10.1093/bmb/ldw056 28104630 PMC5873731

[mcn13411-bib-0023] Ministry of Education, Government of Burkina Faso . (2017). *Plan d'action pour le plan sectoriel de l'éducation du Burkina Faso 2017–2020*. https://planipolis.iiep.unesco.org/fr/node/7185

[mcn13411-bib-0024] Nyundo, A. , Manu, A. , Regan, M. , Ismail, A. , Chukwu, A. , Dessie, Y. , Njau, T. , Kaaya, S. F. , & Smith Fawzi, M. C. (2020). Factors associated with depressive symptoms and suicidal ideation and behaviors amongst sub‐Saharan African adolescents aged 10‐19 years: A cross‐sectional study. Tropical Medicine and International Health, 25(1), 54–69. 10.1111/tmi.13336 31698526

[mcn13411-bib-0025] Patton, G. C. , Neufeld, L. M. , Dogra, S. , Frongillo, E. A. , Hargreaves, D. , He, S. , Mates, E. , Menon, P. , Naguib, M. , & Norris, S. A. (2021). Nourishing our future: The Lancet Series on adolescent nutrition. Lancet, 399(10320), 123–125. 10.1016/S0140-6736(21)02140-1 34856189

[mcn13411-bib-0026] Patton, G. C. , Sawyer, S. M. , Santelli, J. S. , Ross, D. A. , Afifi, R. , Allen, N. B. , Arora, M. , Azzopardi, P. , Baldwin, W. , Bonell, C. , Kakuma, R. , Kennedy, E. , Mahon, J. , McGovern, T. , Mokdad, A. H. , Patel, V. , Petroni, S. , Reavley, N. , Taiwo, K. , … Viner, R. M. (2016). Our future: A Lancet commission on adolescent health and well‐being. Lancet, 387, 2423–2478. 10.1016/S0140-6736(16)00579-1 27174304 PMC5832967

[mcn13411-bib-0027] Population Reference Bureau (PRB) . (2021). World population data sheet. https://interactives.prb.org/2021-wpds/

[mcn13411-bib-0028] Richman, W. L. , Kiesler, S. , Weisband, S. , & Drasgow, F. (1999). A meta‐analytic study of social desirability distortion in computer‐administered questionnaires, traditional questionnaires, and interviews. Journal of Applied Psychology, 84(5), 754–775.

[mcn13411-bib-0029] Southern and Eastern Africa Consortium for Monitoring Educational Quality . (2019). SACMEQ indicators III: Main study. SACMEQ.

[mcn13411-bib-0030] UNESCO Institute for Statistics . (2021). Data for the sustainable development goals. Education and literacy. UNESCO. http://uis.unesco.org/en/

[mcn13411-bib-0033] United Nations, Department of Economic and Social Affairs, Population Division . (2015). Population 2030: Demographic challenges and opportunities for sustainable development planning (ST/ESA/SER.A/389). United Nations.

[mcn13411-bib-0034] United Nations, Department of Economic and Social Affairs, Population Division . (2019). World Population Prospects 2019, Online Edition. Review 1.

[mcn13411-bib-0035] United Nations Development Programme . (2018). Human development report 2018. http://hdr.undp.org/en/2018-update

[mcn13411-bib-0036] United Nations Population Fund and Population Reference Bureau . (2012). The status report on adolescents and young people in Sub‐Saharan Africa: Opportunities and challenges. UNFPA.

[mcn13411-bib-0038] United States Agency for International Development . (2021). *The DHS Program: Demographic and health surveys*. www.dhsprogram.com

[mcn13411-bib-0039] World Bank . (2019). Ending learning poverty: What will it take? World Bank. License: CC BY 3.0 IGO.

[mcn13411-bib-0041] World Health Organization . (2019). Non‐communicable diseases and their risk factors. Global School‐based Student Health Survey (GSHS). https://www.who.int/ncds/surveillance/gshs/en/

[mcn13411-bib-0042] Youth in Mind . (2022). *Strengths and Difficulties Questionnaire*. www.sdqinfo.org

