# Supplementary material for: Adolescent health and well‐being in sub‐Saharan Africa: Strengthening knowledge base and research capacity through a collaborative multi‐country school‐based study
Source: Matern Child Nutr. 2023 Mar 31;21(Suppl 1):e13411. doi: 10.1111/mcn.13411 (PMC12208888; doi:10.1111/mcn.13411)
Supplement: Supplementary file 1 — Supplementary information. [file MCN-21-e13411-s001.docx]

**Appendix 1: Ethical approvals**

| **Sr. #** | **Institutional Review Board (IRB)** |
| --- | --- |
| 1 | Harvard Human Research Protection Program, USA  (approval #: IRB19-0822) |
| 2 | Centre de Recherche en Sante de Nouna, Ministere de la Sante, Burkina Faso(approval #: 2019-119/MS/SG/INSP/CRSN) |
| 3 | IRB, Addis Continental Institute of Public Health, Ethiopia  (Approval #: ACIPH/IRB/002/2019) |
| 4 | Biomedical Research Ethics Committee, College of Health Sciences, University of Kwazulu-Natal, South Africa  (Approval #: BE450/19) |
| 5 | Ministry of Education, Sudan  (Approval date: 2019/3/4) |
| 6 | IRB, Ahfad University for Women, Sudan  (Approval date: 2019/30/05) |
| 7 | National Health Research Ethics Review Committee National Institute for Medical Research, Tanzania  (Approval #: NIMR/HQ/R.8c/Vol.I/1507) |

**Appendix 2** ARISE School Health Adolescent Survey Tool

**AA. LOCATION**

Interview date and time automatically coded by ODK.

| **Code** | **Question** | **Responses** |
| --- | --- | --- |
| AA01 | District Name |  |
| AA02 | Sub-District/ Parish/Kebele name |  |
| AA03 | School Name |  |
| AA04 | Individual Identification Number |  |
| AA05 | Data Collectors Code |  |

**A. DEMOGRAPHICS**

A01 What is your date of birth? If you are not sure, give your best guess.

__ __ / __ __ / __ __ __ __ (DD/MM/YYYY)

A02 Ask if not obvious: Sex of the respondent.

Male………………………………….... 0

Female………………………………… 1

A03 In what grade/class/standard are you?

5

6

7

8

Other, specify: _______ 94

A04 Have you done any work in the last 12 months? By work, I mean any activity to earn money or obtain food.

Yes………………………………………... 1

If yes, how many hours per week? _______

No………………………………………… 0

Don’t know…………………………….…. 98

Refuse to answer……………………… 99

A05 Are your mother and father alive?

Both alive………………………………….. 1

Only mother alive……………………… 2

Only father alive……………………….. 3

Both not alive.………………………….. 4

Don’t know…………………………….…. 98

Refuse to answer……………………… 99

A06 Who are you currently living with? (Allow multiple responses)

Mother………...…………………………… 1

Father ……….……………………………... 2

Other male guardian……..………….. 3

Other female guardian …….………. 4

Sibling(s)…………………………………… 5

By myself only………………………….. 6

Other, specify: ____________ 94

Don’t know………………………………. 98

Refuse to answer……………………… 99

A07 What is the highest level of education that your father/male guardian received?

None……………………………………… 0

Primary…………………………………. 1

Secondary ……………………………. 2

Technical/Vocational…………….. 3

University/College…………………. 4

Don’t have father/male guardian 97

Don’t know……………………………. 98

Refuse to answer………………….. 99

A08 What is the occupation of your father/male guardian? *Select all that apply.*

Farmer ……………………………………. 1

Merchant…………………………………. 2

Teacher…………………………………… 3

Government Worker…………….… 4

Unemployed……………………………. 5

Other, specify: ___________ 94

Don’t have father/male guardian 97

Don’t know……………………………. 98

Refuse to answer………………….. 99

A09 What is the highest level of education that your mother/female guardian received?

None……………………………………… 0

Primary …………………………….…… 1

Secondary …………………………….. 2

Technical/Vocational…………….. 3

University/College………………….. 4

Don’t have mother/female guardian 97

Don’t know……………………………. 98

Refuse to answer………………….. 99

A10 What is the occupation of your mother/female guardian? *Select all that apply.*

Farmer ………………………………….. 1

Merchant………………………………… 2

Teacher………………………………….. 3

Other Government Worker…….. 4

Unemployed……………………………. 5

Homemaker…………………………….. 6

Other, specify: ___________ 94

Don’t have mother/female guardian 97

Don’t know……………………………. 98

Refuse to answer………………….. 99

A11 How many brother or sisters (including step- and half-brothers and sisters) live with you at home?

Number of siblings: ________

Don’t know……………………………. 98

Refuse to answer………………….. 99

A12 How long does it usually take you to get to school from your home?

Less than 15 minutes………………… 1

15-30 minutes…………………………… 2

30-45 minutes………………………….. 3

45-60 minutes………………………….. 4

More than 60 minutes……………… 5

Don’t know……………………………. 98

Refuse to answer………………….. 99

A13 How do you usually get to school?

Walk…………………………………………. 1

Ride a bike……………………………….. 2

Ride the bus…………………………….. 3

Catch a ride with someone………. 4

Take a taxi………………………………… 5

Other, specify: ____________ 94

Don’t know……………………………. 98

Refuse to answer………………….. 99

A14 What time do you usually arrive to school?

Before 8am………………………………. 1

Between 8-10am……………………… 2

Between 10am-noon……………….. 3

After noon……………………………….. 4

Don’t know……………………………. 98

Refuse to answer………………….. 99

A15 What time do you usually leave school?

Before 10am……………………….…… 1

Between 10am-noon……………….. 2

Between noon-2pm…………………. 3

Between 2pm-4pm………………….. 4

After 4pm………………………………… 5

Don’t know……………………………. 98

Refuse to answer………………….. 99

**B. SOCIO-ECONOMY**

B01 What type of fuel does your household mainly use for cooking? *Select all that apply.*

Electricity……………………………….... 1

Gas (LPG, natural gas, or biogas) 2

Kerosene………………………………….. 3

Coal………….………………………………. 4

Wood, straw, grass, or animal dung ……. 5

No food is cooked in the household …….. 6

Other, specify: ___________ 94

Don’t know……………………………. 98

Refuse to answer………………….. 99

B02 How many times did you travel away on holiday/vacation with your family during the past 12 months?

Never……………………………………... 0

Once……………………………………… 1

Twice…………………………………….. 2

Three or more times……………… 3

Don’t know……………………………. 98

Refuse to answer………………….. 99

B03 Does your household have the following items?

Yes No

B03a Electricity?..................................... 1 0

B03b A radio?.......................................... 1 0

B03c A television?.................................. 1 0

B03d A mobile phone?............................ 1 0

B03e A refrigerator?............................... 1 0

B03f A washing machine?..................... 1 0

B03g A computer?................................. 1 0

B03h A photo camera?.......................... 1 0

B03i A DVD/CD player?......................... 1 0

B03j A bed/mattress?............................ 1 0

B03k A table?.......................................... 1 0

B03l A chair?.......................................... 1 0

B03m A cabinet/cupboard?..................... 1 0

B03n A bicycle?...................................... 1 0

B03o A motorcycle or motor scooter?... 1 0

B03p A car or truck?............................... 1 0

B03q A solar panel?................................ 1 0

**C. WATER, SANITATION AND HYGIENE (WASH)**

C01 What is the main source of drinking water in your household?

Water that is piped to my house…………………… 1

Water that is piped to my neighborhood………. 2

Water from a well, spring, river, or lake………… 3

Bottled water………………………………………………... 4

Other, specify: ____________ 94

Don’t know……………………………………………………. 98

Refuse to answer…………………………………………... 99

C02 Does your household do anything to the water to make it safer to drink?

No …………….............................. 0

Yes………………………………………... 1

Don’t know……………………………. 98

Refuse to answer………………….. 99

*If the response is 0 or 98, skip to B4.*

C03 What does your household usually do to make the water safer to drink? *Select all that apply.*

Boil………………………………........... 1

Add bleach/chlorine………………. 2

Strain through a cloth……………. 3

Use water filter……………………… 4

Solar disinfection…………………… 5

Let it stand/settle…………………... 6

Other, specify: ____________ 94

Don’t know……………………………. 98

Refuse to answer………………….. 99

C04 What kind of toilet facility do members of your household usually use?

Flush or pour flush toilet………….. 1

Pit toilet/latrine……………………….. 2

No facility/bush/field……………….. 3

Other, specify: ____________ 94

Don’t know……………………………. 98

Refuse to answer………………….. 99

*If the response is 3, skip to B06.*

C05 Do you share this toilet facility with other households?

No………………………………………....... 0

Yes………………………………………...... 1

Don’t know……………………………. 98

Refuse to answer………………….. 99

The next questions ask about cleaning your teeth and washing your hands.

C06 How many times did you clean or brush your teeth yesterday?

Number of times per day: ________

Don’t know……………………………. 98

Refuse to answer………………….. 99

C07 How many times did you visit the dentist during the past year?

Number of times per year: ________

Don’t know……………………………. 98

Refuse to answer………………….. 99

C08 How often do you usually wash your hands before eating? Please answer with never, rarely, sometimes, most of the time, or always.

Never……………………………………... 0

Rarely…………………………………….. 1

Sometimes……………………………… 2

Most of the time…………………….. 3

Always……………………………………. 4

Don’t know……………………………. 98

Refuse to answer………………….. 99

C09 How often do you usually wash your hands after using the toilet or latrine? Please answer with never, rarely, sometimes, most of the time, or always.

Never……………………………………... 0

Rarely…………………………………….. 1

Sometimes……………………………… 2

Most of the time…………………….. 3

Always……………………………………. 4

Don’t know……………………………. 98

Refuse to answer………………….. 99

C10 What do you usually use to wash your hands at home?

Don’t wash hands at home…… 0

Water only……………………………. 1

Water and soap…………………….. 2

Water and ash, mud, or sand… 3

Other, specify: ____________ 94

Don’t know……………………………. 98

Refuse to answer………………….. 99

**D. ANTIMICROBIAL RESISTANCE**

D01 Have you ever heard of antibiotics?

No............................................ 0

Yes............................................ 1

Don’t know……………………………. 98

Refuse to answer………………….. 99

D02 Have you ever used antibiotics? Most antibiotics are medicines taken orally in capsule form.

No............................................ 0

Yes............................................ 1

Don’t know……………………………. 98

Refuse to answer………………….. 99

*If the response is 0 or 98, skip to D04.*

D03 When did you last use antibiotics?

More than 1 month ago………….. 1

More than 6 months ago………… 2

More than 12 months ago………. 3

Don’t know……………………………. 98

Refuse to answer………………….. 99

D04 When you last used antibiotics, why did you take them? *Select all that apply*.

Cough……………………………………….. 1

Sore throat………………………………. 2

Fever……………………………………….. 3

Runny nose………………………………. 4

Ear ache……………………………………. 5

Rapid or difficult breathing………. 6

Skin problem……………………………. 7

Loss of appetite……………………….. 8

Vomiting………………………………….. 9

Diarrhea…………………………………… 10

Upset stomach…………………………. 11

Malaria…………………………………….. 12

Other, specify: ______________ 94

Don’t know……………………………. 98

Refuse to answer………………….. 99

**E. FEMALE MENSTRUATION**

E01 Have you heard of the term 'menstruation' or having one's 'period'?

No............................................ 0

Yes............................................ 1

Don’t know……………………………. 98

Refuse to answer………………….. 99

*If the response is 98, skip to next section.*

E02 Where did you first learn about menstruation?

At school………………………………….. 1

From a parent or other relative… 2

From a friend…………………………….. 3

From television or magazines……. 4

Other, specify: ___________ 94

Don’t know……………………………. 98

Refuse to answer………………….. 99

*Females 🡪 Answer questions E03-E06.*

*Males 🡪 Skip to next section.*

E03 Have you had your first period?

No……..……………………………………. 0

Yes.…………………………………………. 1

Don’t know……………………………. 98

Refuse to answer………………….. 99

*If the response is 0 or 98, skip to next section.*

E04 What do you mainly use during periods?

Cloth/cloth pads………………….…… 1

Disposable sanitary napkins/pads 2

Tampons/menstrual cups…………. 3

Other, specify: ___________ 94

Don’t know……………………………. 98

Refuse to answer………………….. 99

E05 Have you ever missed school because of your period?

No…………………………………………….. 0

Yes – one time…………………………. 1

Yes – more than once………………. 2

Don’t know……………………………. 98

Refuse to answer………………….. 99

*If the response is 0 or 98, skip to next section.*

E06 What are the reasons you missed school due to your period? *Select all that apply*.

Pain/discomfort………………….…… 1

Embarrassment………………………. 2

Fear of leaking……………..…………. 3

No water at school…………………. 4

No changing room available……. 5

Lack of access to sanitary products 6

Other, specify: ___________ 94

Don’t know……………………………. 98

Refuse to answer………………….. 99

**F. PHYSICAL ACTIVITY**

The next three questions ask about physical activity. Physical activity is any activity that increases your heart rate and makes you breathe hard. Physical activity can be done in sports, playing with friends, or walking to school. Some examples of physical activity are running, fast walking, biking, dancing, football, and [*list other* *examples*].

F01 During the past week, on how many days were you physically active for a total of at least one hour per day? Please add up all the time you spent in any kind of physical activity each day.

0 days…………………………………….. 0

1 day……………………………………... 1

2 days…………………………………….. 2

3 days…………………………………….. 3

4 days…………………………………….. 4

5 days…………………………………….. 5

6 days…………………………………….. 6

7 days…………………………………….. 7

Don’t know……………………………. 98

Refuse to answer………………….. 99

F02 During the past week, on how many days did you walk or ride a bicycle to or from school, work, store, or other location?

0 days…………………………………….. 0

1 day……………………………………... 1

2 days…………………………………….. 2

3 days…………………………………….. 3

4 days…………………………………….. 4

5 days…………………………………….. 5

6 days…………………………………….. 6

7 days…………………………………….. 7

Don’t know……………………………. 98

Refuse to answer………………….. 99

F03 This about this past school year. On average, how many days did you go to a physical education (PE) class each week? If you are not sure, please give your best guess.

________ days each week

Don’t know……………………………. 98

Refuse to answer………………….. 99

**G. EATING BEHAVIORS**

*Breakfast Today*

G01a Did you eat breakfast today?

No............................................ 0

Yes............................................ 1

Don’t know……………………………. 98

Refuse to answer………………….. 99

*If the response is 0 or 98, skip to G02a.*

G01b At what time did you ea breakfast?

Between 6am and 8am……………. 1

Between 8am and 10am……..…… 2

Between 10am and noon…………. 3

Between noon and 3 pm………….. 4

Don’t know……………………………… 98

Refuse to answer…………………….. 99

G01c Where did you eat breakfast?

Home………………………………………. 1

School……………………………………… 2

Restaurant/Food Vendor………… 3

Other, specify: ________ 94

Don’t know…………………………….. 98

Refuse to answer……………………. 99

G01d What did you have for breakfast? *Select all that apply.*

**Dark green leafy vegetables** (*Spinach, romaine lettuce, kale, turnip greens, bok choy, collard, chard, arugula, mustard greens, fresh herbs*)…………………………………………………………………….. 1

**Cruciferous vegetables** (*Broccoli, cauliflower, cabbage, Brussels sprouts, kohlrabi*)… 2

**Deep orange vegetables** (*Carrot, pumpkin, yellow and orange squash*)………………….. 3

**Deep orange fruits** (*Mango, ripe papaya, cantaloupe, apricot*)………………………………… 4

**Deep orange tubers** (*Orange sweet potato*)…………………………………………………………….. 5

**Other vegetables** (*Tomato, pepper, cucumber, onion, eggplant, zucchini, beetroot*).. 6

**Citrus fruits** (*Orange, lemon, grapefruit, mandarin, tangerine [whole fruit, not juices]*) 7

**Other fruits** (*Apple, peach, pear, plum, banana, grapes, berries, melon, guava, avocado [whole fruit, not juices]*)……………………………………………………………………………………………………………………… 8

**Legumes** (*Beans, peas, lentils, pulses, legume-based products [tofu, soymilk] [excludes peanut]*) 9

**Nuts and seeds** (*Includes ground [e.g. peanut] and tree nuts, nut and seed butters/tahini*) 10

**Poultry** (*Chicken, turkey, duck. Excludes luncheon meat, and pâté. Includes organs.*) 11

**Fish** (*Excludes shellfish*)……………………………………………………………………………………………. 12

**Whole grains** (*Breads, cereals, porridges, noodles and products made of cereal flour containing at least 50% wholegrain*)………………………………………………………………………………………………………. 13

**Liquid oils** (*Olive, rapeseed, sunflower, peanut, corn, sesame, etc. Excludes semisolid oils [e.g. coconut and palm oil*]……………………………………………………………………………………………………………. 14

**Low fat dairy** (*Milk, cheese, yogurt, kefir, containing 2% or less m.f.*)………………………. 15

**Eggs**………………………………………………………………………………………………………………………….. 16

**White roots and tubers** (*White, yellow, red potato, yam (white), cassava, tapioca, white/beige sweet potato.*)……………………………………………………………………………………………………………………. 17

**Red meat** (*Beef, pork, goat, or lamb/mutton. Includes organs.*)………………………………. 18

**Processed meat** (*Sausages, salami, bologna, hot dogs, bacon, pâté, luncheon meat*) 19

**Refined grains and baked goods** (*Breads, ready-to-eat breakfast cereals, porridges, noodles and products made of flour containing refined grains only [e.g. white pasta, rice, bread, baked goods]*) 20

**Sugar-sweetened beverages** (*Soft drinks, energy and sports drinks. Excludes sugar-added fruit nectars, milk or cereal based sugary drinks, fruit syrups, juices*)……………………………………………. 21

**Sweets and ice cream** (*Candy, chocolate, cake, cookie, sugar cane, ice cream, including homemade*) 22

**High fat dairy** (*Milk, cheese, yogurt, kefir, containing more than 2% m.f., butter, cream, cheese*) 23

**Juice** (*Any juice regardless of fruit content or sugar content. Includes nectars, aguas frescas.*) 24

**Fried foods eaten away from home** (*Street food, fast food, eating out*)…………………… 25

Other, specify: ________ 94

Don’t know…………………………*…………………………………………………………………………..*  98

Refuse to answer…………………..*………………………………………………………………………..*  99

G01e Who usually prepares your breakfast?

Don’t usually eat breakfast………. 0

Parents…………………………………….. 1

School canteen…………………………. 2

Food vendor on school campus… 3

Food vendor outside of school campus 4

I prepare it myself……………………… 5

Other, specify: ___________ 94

Don’t know………………………………… 98

Refuse to answer……………………….. 99

*Lunch Today/Yesterday*

G02a If the interview is being conducted before lunchtime, ask: Did you have lunch yesterday?

If the interview is being conducted after lunchtime, ask: Did you have lunch today?

No................................................ 0

Yes............................................... 1

Don’t know……………………………. 98

Refuse to answer………………….. 99

*If the response is 0 or 98, skip to G02d.*

G02b At what time did you have lunch?

Before 10am……..…………………….. 1

Between 10am and noon…………. 2

Between noon and 3 pm………….. 3

Between 3pm and 6pm……………. 4

After 6pm………………………………… 5

Don’t know……………………………. 98

Refuse to answer………………….. 99

G02c Where did you eat lunch?

Home…………………………………….. 1

School……………………………………. 2Restaurant/Food Vendor………… 3

Other, specify: ________ 94

Don’t know……………………………. 98

Refuse to answer………………….. 99

G02d What did you have for lunch? *Select all that apply.*

**Dark green leafy vegetables** (*Spinach, romaine lettuce, kale, turnip greens, bok choy, collard, chard, arugula, mustard greens, fresh herbs*)…………………………………………………………………….. 1

**Cruciferous vegetables** (*Broccoli, cauliflower, cabbage, Brussels sprouts, kohlrabi*)… 2

**Deep orange vegetables** (*Carrot, pumpkin, yellow and orange squash*)………………….. 3

**Deep orange fruits** (*Mango, ripe papaya, cantaloupe, apricot*)………………………………… 4

**Deep orange tubers** (*Orange sweet potato*)…………………………………………………………….. 5

**Other vegetables** (*Tomato, pepper, cucumber, onion, eggplant, zucchini, beetroot*).. 6

**Citrus fruits** (*Orange, lemon, grapefruit, mandarin, tangerine [whole fruit, not juices]*) 7

**Other fruits** (*Apple, peach, pear, plum, banana, grapes, berries, melon, guava, avocado [whole fruit, not juices]*)……………………………………………………………………………………………………………………… 8

**Legumes** (*Beans, peas, lentils, pulses, legume-based products [tofu, soymilk] [excludes peanut]*) 9

**Nuts and seeds** (*Includes ground [e.g. peanut] and tree nuts, nut and seed butters/tahini*) 10

**Poultry** (*Chicken, turkey, duck. Excludes luncheon meat, and pâté. Includes organs.*) 11

**Fish** (*Excludes shellfish*)……………………………………………………………………………………………. 12

**Whole grains** (*Breads, cereals, porridges, noodles and products made of cereal flour containing at least 50% wholegrain*)………………………………………………………………………………………………………. 13

**Liquid oils** (*Olive, rapeseed, sunflower, peanut, corn, sesame, etc. Excludes semisolid oils [e.g. coconut and palm oil*]……………………………………………………………………………………………………………. 14

**Low fat dairy** (*Milk, cheese, yogurt, kefir, containing 2% or less m.f.*)………………………. 15

**Eggs**………………………………………………………………………………………………………………………….. 16

**White roots and tubers** (*White, yellow, red potato, yam (white), cassava, tapioca, white/beige sweet potato.*)……………………………………………………………………………………………………………………. 17

**Red meat** (*Beef, pork, goat, or lamb/mutton. Includes organs.*)………………………………. 18

**Processed meat** (*Sausages, salami, bologna, hot dogs, bacon, pâté, luncheon meat*) 19

**Refined grains and baked goods** (*Breads, ready-to-eat breakfast cereals, porridges, noodles and products made of flour containing refined grains only [e.g. white pasta, rice, bread, baked goods]*) 20

**Sugar-sweetened beverages** (*Soft drinks, energy and sports drinks. Excludes sugar-added fruit nectars, milk or cereal based sugary drinks, fruit syrups, juices*)……………………………………………. 21

**Sweets and ice cream** (*Candy, chocolate, cake, cookie, sugar cane, ice cream, including homemade*) 22

**High fat dairy** (*Milk, cheese, yogurt, kefir, containing more than 2% m.f., butter, cream, cheese*) 23

**Juice** (*Any juice regardless of fruit content or sugar content. Includes nectars, aguas frescas.*) 24

**Fried foods eaten away from home** (*Street food, fast food, eating out*)…………………… 25

Other, specify: ________ 94

Don’t know…………………………*…………………………………………………………………………..*  98

Refuse to answer…………………..*………………………………………………………………………..*  99

G02e Who usually prepares your lunch?

Don’t usually eat lunch……………. 0

Parents…………………………………….. 1

School canteen…………………………. 2

Food vendor on school campus… 3

Food vendor outside of school campus 4

I prepare it myself……………………… 5

Other, specify: ___________ 94

Don’t know………………………………… 98

Refuse to answer……………………….. 99

*Dinner Yesterday*

G03a Did you have dinner yesterday?

No............................................ 0

Yes............................................ 1

Don’t know……………………………. 98

Refuse to answer………………….. 99

*If the response is 0 or 98, skip to G04a.*

G03b At what time did you have dinner?

Between 3pm and 6pm………… 1

Between 6pm and 9pm………… 2

Between 9pm and midnight… 3

Don’t know……………………………. 98

Refuse to answer………………….. 99

G03c Where did you eat dinner?

Home…………………………………….. 1

School……………………………………. 2

Restaurant/Food Vendor………… 3

Other, specify: ________ 94

Don’t know……………………………. 98

Refuse to answer………………….. 99

G03d What did you eat for dinner? *Select all that apply.*

**Dark green leafy vegetables** (*Spinach, romaine lettuce, kale, turnip greens, bok choy, collard, chard, arugula, mustard greens, fresh herbs*)…………………………………………………………………….. 1

**Cruciferous vegetables** (*Broccoli, cauliflower, cabbage, Brussels sprouts, kohlrabi*)… 2

**Deep orange vegetables** (*Carrot, pumpkin, yellow and orange squash*)………………….. 3

**Deep orange fruits** (*Mango, ripe papaya, cantaloupe, apricot*)………………………………… 4

**Deep orange tubers** (*Orange sweet potato*)…………………………………………………………….. 5

**Other vegetables** (*Tomato, pepper, cucumber, onion, eggplant, zucchini, beetroot*).. 6

**Citrus fruits** (*Orange, lemon, grapefruit, mandarin, tangerine [whole fruit, not juices]*) 7

**Other fruits** (*Apple, peach, pear, plum, banana, grapes, berries, melon, guava, avocado [whole fruit, not juices]*)……………………………………………………………………………………………………………………… 8

**Legumes** (*Beans, peas, lentils, pulses, legume-based products [tofu, soymilk] [excludes peanut]*) 9

**Nuts and seeds** (*Includes ground [e.g. peanut] and tree nuts, nut and seed butters/tahini*) 10

**Poultry** (*Chicken, turkey, duck. Excludes luncheon meat, and pâté. Includes organs.*) 11

**Fish** (*Excludes shellfish*)……………………………………………………………………………………………. 12

**Whole grains** (*Breads, cereals, porridges, noodles and products made of cereal flour containing at least 50% wholegrain*)………………………………………………………………………………………………………. 13

**Liquid oils** (*Olive, rapeseed, sunflower, peanut, corn, sesame, etc. Excludes semisolid oils [e.g. coconut and palm oil*]……………………………………………………………………………………………………………. 14

**Low fat dairy** (*Milk, cheese, yogurt, kefir, containing 2% or less m.f.*)………………………. 15

**Eggs**………………………………………………………………………………………………………………………….. 16

**White roots and tubers** (*White, yellow, red potato, yam (white), cassava, tapioca, white/beige sweet potato.*)……………………………………………………………………………………………………………………. 17

**Red meat** (*Beef, pork, goat, or lamb/mutton. Includes organs.*)………………………………. 18

**Processed meat** (*Sausages, salami, bologna, hot dogs, bacon, pâté, luncheon meat*) 19

**Refined grains and baked goods** (*Breads, ready-to-eat breakfast cereals, porridges, noodles and products made of flour containing refined grains only [e.g. white pasta, rice, bread, baked goods]*) 20

**Sugar-sweetened beverages** (*Soft drinks, energy and sports drinks. Excludes sugar-added fruit nectars, milk or cereal based sugary drinks, fruit syrups, juices*)……………………………………………. 21

**Sweets and ice cream** (*Candy, chocolate, cake, cookie, sugar cane, ice cream, including homemade*) 22

**High fat dairy** (*Milk, cheese, yogurt, kefir, containing more than 2% m.f., butter, cream, cheese*) 23

**Juice** (*Any juice regardless of fruit content or sugar content. Includes nectars, aguas frescas.*) 24

**Fried foods eaten away from home** (*Street food, fast food, eating out*)…………………… 25

Other, specify: ________ 94

Don’t know…………………………*…………………………………………………………………………..*  98

Refuse to answer…………………..*………………………………………………………………………..*  99

G03e Who usually prepares your dinner?

Don’t usually eat dinner……………. 0

Parents…………………………………….. 1

School canteen…………………………. 2

Food vendor on school campus… 3

Food vendor outside of school campus 4

I prepare it myself……………………… 5

Other, specify: ___________ 94

Don’t know………………………………… 98

Refuse to answer……………………….. 99

*Snacks*

G04a Yesterday during the day and night, did you eat anything between the meals?

Yes............................................ 1

No............................................ 0

Don’t know……………………………. 98

Refuse to answer………………….. 99

*If the response is 0 or 98, skip to G05.*

G04b What did you eat? *Select all that apply.*

**Dark green leafy vegetables** (*Spinach, romaine lettuce, kale, turnip greens, bok choy, collard, chard, arugula, mustard greens, fresh herbs*)…………………………………………………………………….. 1

**Cruciferous vegetables** (*Broccoli, cauliflower, cabbage, Brussels sprouts, kohlrabi*)… 2

**Deep orange vegetables** (*Carrot, pumpkin, yellow and orange squash*)………………….. 3

**Deep orange fruits** (*Mango, ripe papaya, cantaloupe, apricot*)………………………………… 4

**Deep orange tubers** (*Orange sweet potato*)…………………………………………………………….. 5

**Other vegetables** (*Tomato, pepper, cucumber, onion, eggplant, zucchini, beetroot*).. 6

**Citrus fruits** (*Orange, lemon, grapefruit, mandarin, tangerine [whole fruit, not juices]*) 7

**Other fruits** (*Apple, peach, pear, plum, banana, grapes, berries, melon, guava, avocado [whole fruit, not juices]*)……………………………………………………………………………………………………………………… 8

**Legumes** (*Beans, peas, lentils, pulses, legume-based products [tofu, soymilk] [excludes peanut]*) 9

**Nuts and seeds** (*Includes ground [e.g. peanut] and tree nuts, nut and seed butters/tahini*) 10

**Poultry** (*Chicken, turkey, duck. Excludes luncheon meat, and pâté. Includes organs.*) 11

**Fish** (*Excludes shellfish*)……………………………………………………………………………………………. 12

**Whole grains** (*Breads, cereals, porridges, noodles and products made of cereal flour containing at least 50% wholegrain*)………………………………………………………………………………………………………. 13

**Liquid oils** (*Olive, rapeseed, sunflower, peanut, corn, sesame, etc. Excludes semisolid oils [e.g. coconut and palm oil*]……………………………………………………………………………………………………………. 14

**Low fat dairy** (*Milk, cheese, yogurt, kefir, containing 2% or less m.f.*)………………………. 15

**Eggs**………………………………………………………………………………………………………………………….. 16

**White roots and tubers** (*White, yellow, red potato, yam (white), cassava, tapioca, white/beige sweet potato.*)……………………………………………………………………………………………………………………. 17

**Red meat** (*Beef, pork, goat, or lamb/mutton. Includes organs.*)………………………………. 18

**Processed meat** (*Sausages, salami, bologna, hot dogs, bacon, pâté, luncheon meat*) 19

**Refined grains and baked goods** (*Breads, ready-to-eat breakfast cereals, porridges, noodles and products made of flour containing refined grains only [e.g. white pasta, rice, bread, baked goods]*) 20

**Sugar-sweetened beverages** (*Soft drinks, energy and sports drinks. Excludes sugar-added fruit nectars, milk or cereal based sugary drinks, fruit syrups, juices*)……………………………………………. 21

**Sweets and ice cream** (*Candy, chocolate, cake, cookie, sugar cane, ice cream, including homemade*) 22

**High fat dairy** (*Milk, cheese, yogurt, kefir, containing more than 2% m.f., butter, cream, cheese*) 23

**Juice** (*Any juice regardless of fruit content or sugar content. Includes nectars, aguas frescas.*) 24

**Fried foods eaten away from home** (*Street food, fast food, eating out*)…………………… 25

Other, specify: ________ 94

Don’t know…………………………*…………………………………………………………………………..*  98

Refuse to answer…………………..*………………………………………………………………………..*  99

G04c Who usually prepares your snacks? *Select all that apply*.

Don’t usually eat snacks……………. 0

Parents…………………………………….. 1

School canteen…………………………. 2

Food vendor on school campus… 3

Food vendor outside of school campus 4

I prepare it myself……………………… 5

Other, specify: ___________ 94

Don’t know………………………………… 98

Refuse to answer……………………….. 99

**H. DIETARY QUALITY**

H01 *Prime Diet Quality Score (PDQS)*

How often did you eat the following foods over the last week? If you are not sure, give your best guess.

|  | Details/examples of foods | 0  Never | 1  Once/ week | 2  2-4 times/ week | 3  5-7 times/ week | 4  ≥ 1 time/ day | 98  Don’t know | 97  Refuse to answer |
| --- | --- | --- | --- | --- | --- | --- | --- | --- |
| Group 1 components | |  |  |  |  |  |  |  |
| Dark green leafy vegetables | Spinach, romaine lettuce, kale, turnip greens, bok choy, collard, chard, arugula, mustard greens, fresh herbs |  |  |  |  |  |  |  |
| Cruciferous vegetables | Broccoli, cauliflower, cabbage, Brussels sprouts, kohlrabi |  |  |  |  |  |  |  |
| Deep orange vegetables | Carrot, pumpkin, yellow and orange squash |  |  |  |  |  |  |  |
| Deep orange fruits | Mango, ripe papaya, cantaloupe, apricot |  |  |  |  |  |  |  |
| Deep orange tubers | Orange sweet potato |  |  |  |  |  |  |  |
| Other vegetables | Tomato, pepper, cucumber, onion, eggplant, zucchini, beetroot |  |  |  |  |  |  |  |
| Citrus fruits | Orange, lemon, grapefruit, mandarin, tangerine (whole fruit, not juices) |  |  |  |  |  |  |  |
| Other fruits | Apple, peach, pear, plum, banana, grapes, berries, melon, guava, avocado (whole fruit, not juices) |  |  |  |  |  |  |  |
| Legumes | Beans, peas, lentils, pulses, legume-based products (tofu, soymilk) (excludes peanut) |  |  |  |  |  |  |  |
| Nuts and seeds | Includes ground (e.g. peanut) and tree nuts, nut and seed butters/tahini |  |  |  |  |  |  |  |
| Poultry | Excludes luncheon meat, and pâté. Includes organs. |  |  |  |  |  |  |  |
| Fish | Excludes shellfish |  |  |  |  |  |  |  |
| Whole grains | Breads, cereals, porridges, noodles and products made of cereal flour containing at least 50% wholegrain (alternately: fiber:carbohydrate >.1). |  |  |  |  |  |  |  |
| Liquid oils | Olive, rapeseed, sunflower, peanut, corn, sesame, etc. Excludes semisolid oils (e.g. coconut and palm oil) |  |  |  |  |  |  |  |
| Low fat dairy | Milk, cheese, yogurt, kefir, containing 2% or less m.f. |  |  |  |  |  |  |  |
| Eggs |  |  |  |  |  |  |  |  |
| Group 2 components | |  |  |  |  |  |  |  |
| White roots and tubers | White, yellow, red potato, yam (white), cassava, tapioca, white/beige sweet potato. |  |  |  |  |  |  |  |
| Red meat | Beef, pork, goat, or lamb/mutton. Includes organs. |  |  |  |  |  |  |  |
| Processed meat | Sausages, salami, bologna, hot dogs, bacon, pâté, luncheon meat |  |  |  |  |  |  |  |
| Refined grains and baked goods | Breads, pan dulce, ready-to-eat breakfast cereals, porridges, noodles and products made of flour containing refined grains only (e.g. white pasta, rice, bread, baked goods) (alternately: fiber:carbohydrate <.1). |  |  |  |  |  |  |  |
| Sugar-sweetened beverages | Soft drinks, energy and sports drinks. Excludes sugar-added fruit nectars, milk or cereal based sugary drinks, fruit syrups, juices |  |  |  |  |  |  |  |
| Sweets and ice cream | Candy, chocolate, cake, cookie, sugar cane, ice cream, including homemade ones |  |  |  |  |  |  |  |
| High fat dairy | Milk, cheese, yogurt, kefir, containing more than 2% m.f., butter, cream, cheese |  |  |  |  |  |  |  |
| Juice | Any juice regardless of fruit content or sugar content. Includes nectars, aguas frescas. |  |  |  |  |  |  |  |
| "Double-counted" components | |  |  |  |  |  |  |  |
| Fried foods eaten away from home | Street food, fast food, eating out |  |  |  |  |  |  |  |

**I. FOOD SECURITY**

I01a In the past 30 days, was there ever no food to eat of any kind in your house because of lack of resources to get food?

No............................................ 0

Yes............................................ 1

Don’t know……………………………. 98

Refuse to answer………………….. 99

*If the response is 0 or 98, skip to I02a.*

I01b How often did this happen in the past 30 days?

Rarely (1-2 times)………………….. 1

Sometimes (3-10 times)…………. 2

Often (more than 10 times)……. 3

Don’t know……………………………. 98

Refuse to answer………………….. 99

I02a In the past 30 days, did you or any household member go to sleep at night hungry because there was not enough food?

No............................................ 0

Yes............................................ 1

Don’t know……………………………. 98

Refuse to answer………………….. 99

*If the response is 0 or 98, skip to I03a.*

I02b How often did this happen in the past 30 days?

Rarely (1-2 times)………………….. 1

Sometimes (3-10 times)…………. 2

Often (more than 10 times)……. 3

Don’t know……………………………. 98

Refuse to answer………………….. 99

I03a In the past 30 days, did you or any household member go a whole day and night without eating anything at all because there was not enough food?

No............................................ 0

Yes............................................ 1

Don’t know……………………………. 98

Refuse to answer………………….. 99

*If the response is 0 or 98, skip to next section.*

I03b How often did this happen in the past 30 days?

Rarely (1-2 times)………………….. 1

Sometimes (3-10 times)…………. 2

Often (more than 10 times)……. 3

Don’t know……………………………. 98

Refuse to answer………………….. 99

**J. SOCIO-EMOTIONAL DEVELOPMENT**

I am now going to read a set of statements to you. Please answer with “Not True,” “Somewhat True,” or “Certainly True,” as they apply to you over the last six months. It would help us if you answered all items as best you can even if you are not absolutely certain.

J01 I try to be nice to other people. I care about their feelings.

Not true…………………………… 0

Somewhat true……………….. 1

Certainly true………………….. 2

Don’t know……………………………. 98

Refuse to answer………………….. 99

J02 I am restless, I cannot stay still for long.

Not true…………………………… 0

Somewhat true……………….. 1

Certainly true………………….. 2

Don’t know……………………………. 98

Refuse to answer………………….. 99

J03 I get a lot of headaches, stomach-aches or sickness.

Not true…………………………… 0

Somewhat true……………….. 1

Certainly true………………….. 2

Don’t know……………………………. 98

Refuse to answer………………….. 99

J04 I usually share with others, for example: CDs, games, food.

Not true…………………………… 0

Somewhat true……………….. 1

Certainly true………………….. 2

Don’t know……………………………. 98

Refuse to answer………………….. 99

J05 I get very angry and often lose my temper.

Not true…………………………… 0

Somewhat true……………….. 1

Certainly true………………….. 2

Don’t know……………………………. 98

Refuse to answer………………….. 99

J06 I would rather be alone than with people of my age.

Not true…………………………… 0

Somewhat true……………….. 1

Certainly true………………….. 2

Don’t know……………………………. 98

Refuse to answer………………….. 99

J07 I usually do as I am told.

Not true…………………………… 0

Somewhat true……………….. 1

Certainly true………………….. 2

Don’t know……………………………. 98

Refuse to answer………………….. 99

J08 I worry a lot.

Not true…………………………… 0

Somewhat true……………….. 1

Certainly true………………….. 2

Don’t know……………………………. 98

Refuse to answer………………….. 99

J09 I am helpful if someone is hurt, upset, or feeling ill.

Not true…………………………… 0

Somewhat true……………….. 1

Certainly true………………….. 2

Don’t know……………………………. 98

Refuse to answer………………….. 99

J10 I am constantly fidgeting or squirming.

Not true…………………………… 0

Somewhat true……………….. 1

Certainly true………………….. 2

Don’t know……………………………. 98

Refuse to answer………………….. 99

J11 I have one good friend or more.

Not true…………………………… 0

Somewhat true……………….. 1

Certainly true………………….. 2

Don’t know……………………………. 98

Refuse to answer………………….. 99

J12 I fight a lot. I can make other people do what I want.

Not true…………………………… 0

Somewhat true……………….. 1

Certainly true………………….. 2

Don’t know……………………………. 98

Refuse to answer………………….. 99

J13 I am often unhappy, depressed or tearful.

Not true…………………………… 0

Somewhat true……………….. 1

Certainly true………………….. 2

Don’t know……………………………. 98

Refuse to answer………………….. 99

J14 Other people my age generally like me.

Not true…………………………… 0

Somewhat true……………….. 1

Certainly true………………….. 2

Don’t know……………………………. 98

Refuse to answer………………….. 99

J15 I am easily distracted. I find it difficult to concentrate.

Not true…………………………… 0

Somewhat true……………….. 1

Certainly true………………….. 2

Don’t know……………………………. 98

Refuse to answer………………….. 99

J16 I am nervous in new situations. I easily lose confidence.

Not true…………………………… 0

Somewhat true……………….. 1

Certainly true………………….. 2

Don’t know……………………………. 98

Refuse to answer………………….. 99

J17 I am kind to younger children.

Not true…………………………… 0

Somewhat true……………….. 1

Certainly true………………….. 2

Don’t know……………………………. 98

Refuse to answer………………….. 99

J18 I am often accused of lying or cheating.

Not true…………………………… 0

Somewhat true……………….. 1

Certainly true………………….. 2

Don’t know……………………………. 98

Refuse to answer………………….. 99

J19 Other children or young people pick on or bully me.

Not true…………………………… 0

Somewhat true……………….. 1

Certainly true………………….. 2

Don’t know……………………………. 98

Refuse to answer………………….. 99

J20 I often volunteer to help others (parents, teachers, children).

Not true…………………………… 0

Somewhat true……………….. 1

Certainly true………………….. 2

Don’t know……………………………. 98

Refuse to answer………………….. 99

J21 I think before I do things.

Not true…………………………… 0

Somewhat true……………….. 1

Certainly true………………….. 2

Don’t know……………………………. 98

Refuse to answer………………….. 99

J22 I take things that are not mine from home, school, or elsewhere.

Not true…………………………… 0

Somewhat true……………….. 1

Certainly true………………….. 2

Don’t know……………………………. 98

Refuse to answer………………….. 99

J23 I get along better with adults than with people my own age.

Not true…………………………… 0

Somewhat true……………….. 1

Certainly true………………….. 2

Don’t know……………………………. 98

Refuse to answer………………….. 99

J24 I have many fears; I am easily scared.

Not true…………………………… 0

Somewhat true……………….. 1

Certainly true………………….. 2

Don’t know……………………………. 98

Refuse to answer………………….. 99

J25 I finish the work I’m doing. My attention is good.

Not true…………………………… 0

Somewhat true……………….. 1

Certainly true………………….. 2

Don’t know……………………………. 98

Refuse to answer………………….. 99

J26 Have you ever been in a physical fight with someone?

Yes............................................ 1

No............................................ 0

Don’t know/refuse to answer…… 98

*If the response is 0 or 98, skip to J28.*

J27 How often did you get into a physical fight?

Once……………………………………. 1

A few times…………………………. 2

Many times…………………………. 3

All the time…………………………. 4

Don’t know/refuse to answer…… 98

J28 How many close friends (boys and/or girls) do you have? By close friends, we mean those that you can talk about feelings and share secrets with.

J28a Male friends

0 …………………………………….. 0

1 ……………………………………... 1

2 …………………………………….. 2

3 …………………………………….. 3

4 …………………………………….. 4

5 …………………………………….. 5

6 or more………………………… 6

Don’t know……………………………. 98

Refuse to answer………………….. 99

J28b Female friends

0 …………………………………….. 0

1 ……………………………………... 1

2 …………………………………….. 2

3 …………………………………….. 3

4 …………………………………….. 4

5 …………………………………….. 5

6 or more………………………… 6

Don’t know……………………………. 98

Refuse to answer………………….. 99

*If the response is 0 for J01a and J01b, skip to G03a.*

**K. EDUCATIONAL OUTCOMES AND LEARNING RESOURCES**

*Connectedness and Aspirations*

The following questions are about your experience with school.

K01 Do you feel that there is an adult (a teacher or someone else) in school who really cares about you?

No, not at all………………………. 0

No, not much……………………… 1

Yes, some of the time…………. 2

Yes, most of the time…………. 3

Don’t know……………………………. 98

Refuse to answer………………….. 99

K02 During the past month, how many days did you miss school for any reason except for when school was closed or for holidays?

0 days………………………………….. 0

1-2 days………………………………. 1

3-5 days……………………………….. 2

More than 5 days…………………. 3

Don’t know……………………………. 98

Refuse to answer………………….. 99

*If the response is 0, skip to next section.*

If you are a boy and you missed school at least once in the past month:

K03a What were the main reasons you missed school last month? *Select all that apply*.

I was sick……………………………………………….. 1

Lack of school fees…………………………………. 2

Helping out at home………………………………. 3

Babysitting younger brothers or sisters…. 4

Working on the family farm……………………. 5

Working to earn money………………………….. 6

Hanging out with friends………………………… 8

Studying for an exam……………………………… 9

Too tired to go to school………………………… 10

Scared of peers/teacher………………………… 11

Other, specify: ____________ 94

Don’t know……………………………………………. 98

Refuse to answer…………………………………… 99

If you are a girl and you missed school at least once in the past month:

K03b What were the main reasons you missed school last month? Select all that apply.

I was sick……………………………………………….. 1

Lack of school fees…………………………………. 2

Helping out at home………………………………. 3

Babysitting younger brothers or sisters…. 4

Working on the family farm……………………. 5

Working to earn money………………………….. 6

Hanging out with friends………………………… 8

Studying for an exam……………………………… 9

Too tired to go to school………………………… 10

Scared of peers/teacher………………………… 11

Had my period……………………………………….. 12

Other, specify: ____________ 94

Don’t know……………………………………………. 98

Refuse to answer…………………………………… 99

*Educational Outcomes*

K04 How many times have you repeated a grade since you have been in school?

Never……………………………………. 0

Once…………………………………….. 1

Twice……………………………………. 2

Three or more times…………….. 3

Don’t know……………………………. 98

Refuse to answer………………….. 99

*Learning Resources*

K05 How many books do you have at home? If you are not sure, please give your best guess.

Number of books: ________

Don’t know……………………………. 98

Refuse to answer………………….. 99

**L. MEDIA**

The following questions are about your access to media. For example: TV, radio, movies, computers, Internet, and mobile phones.

*Access to Media*

For each item, please tell me whether you have access to it.

L01a Computer, laptop, or tablet (e.g. iPad).

No............................................ 0

Yes............................................ 1

Don’t know……………………………. 98

Refuse to answer………………….. 99

L01b Your own cell or mobile phone.

No............................................ 0

Yes............................................ 1

Don’t know……………………………. 98

Refuse to answer………………….. 99

L01c Someone else’s cell or mobile phone.

No............................................ 0

Yes............................................ 1

Don’t know……………………………. 98

Refuse to answer………………….. 99

L01d Social media account or text/chat account such as Facebook, Twitter, Instagram, WhatsApp, or [*insert other site-specific examples*].

No............................................ 0

Yes............................................ 1

Don’t know……………………………. 98

Refuse to answer………………….. 99

L01e Internet at home.

No............................................ 0

Yes............................................ 1

Don’t know……………………………. 98

Refuse to answer………………….. 99

**M. MENTAL HEALTH**

I would now like to ask you a few questions about your feelings. For each of the following five questions, please answer with hardly ever, much of the time, most of the time or all of the time

Over the last week, how frequently have you experienced the following items?

M01 Low mood, sadness, feeling blah or down, depressed, just can’t be bothered.

Hardly ever ……………………………… 0

Some of the time ……………………. 1

Most of the time …………………….. 2

All of the time………………………….. 3

Don’t know……………………………. 98

Refuse to answer………………….. 99

M02 Feelings of worthlessness, hopelessness, letting people down, or not being a good person.

Hardly ever ……………………………… 0

M Some uch of the time ……………………. 1

Most of the time …………………….. 2

All of the time………………………….. 3

Don’t know……………………………. 98

Refuse to answer………………….. 99

M03 Feeling tired, feeling fatigued, low in energy, hard to get motivated, have to push to get things done, want to rest or lie down a lot.

Hardly ever ……………………………… 0

Some of the time ……………………. 1

Most of the time …………………….. 2

All of the time………………………….. 3

Don’t know……………………………. 98

Refuse to answer………………….. 99

M04 Feeling that life is not very much fun, not feeling good when usually (before getting sick) would feel good, not getting as much pleasure from fun things as usual (before getting sick).

Hardly ever ……………………………… 0

Some of the time ……………………. 1

Most of the time …………………….. 2

All of the time………………………….. 3

Don’t know……………………………. 98

Refuse to answer………………….. 99

M05 Feeling worried, nervous, panicky, tense, keyed up, or anxious.

Hardly ever ……………………………… 0

Some of the time ……………………. 1

Most of the time …………………….. 2

All of the time………………………….. 3

Don’t know……………………………. 98

Refuse to answer………………….. 99

M06 Thoughts or plans about suicide or self-harm.

Hardly ever ……………………………… 0

Some of the time ……………………. 1

Most of the time …………………….. 2

All of the time………………………….. 3

Don’t know……………………………. 98

Refuse to answer………………….. 99

**N. ANTHROPOMETRY**

We are almost at the end of the questionnaire. I am now going to take your height and weight measurements.

N01 Interviewer observes: Clothing worn

None…………………………………… 0

Very light……………………………. 1

Light……………………………………. 2

Medium………………………………. 3

Heavy………………………………….. 4

Not sure……………………………… 98

N02 Interviewer observes: Head cover, hair style, or other item worn on the head and unwilling to remove.

No............................................ 0

Yes............................................ 1

Don’t know .............................. 98

N03a Weight 1 in kg (measure to 1 decimal point).

|__|__|__|.|__|

Refuse to participate……………….. 98

N03b Weight 2 in kg (measure to 1 decimal point).

|__|__|__|.|__|

Refuse to participate……………….. 98

N03c Average of N03a and N03b.

|__|__|__|.|__|

N04a Height 1 in cm (measure to 1 decimal point).

|__|__|__|.|__|

Refuse to participate……………….. 98

N04b Height 2 in cm (measure to 1 decimal point).

|__|__|__|.|__|

Refuse to participate……………….. 98

N04c Average of N01f and N01g.

|__|__|__|.|__|

N05 Body mass index. Divide N01e by N01h.

|__|__|__|.|__|

Refuse to participate……………….. 98

Now I am are going to take your hemoglobin level to measure your level of anemia using this HemoCue machine. I’m going to take a small blood sample from your middle finger. You may feel a slight prick, but the pain should not last long. Are you ready to begin?

N01j Administer the HemoCue measurement.

|__|__|.|__| g/dL

Refuse to participate……………….. 98

**Appendix 3: Checklist for scheduled and unscheduled events in targeted grades**

Name of the school:

Grade and section:

Month and year:

Note: The schedule should be filled out in consultation with the school principal and/or class teacher.

| Sr. # | Description | Week 1 | Week 2 | Week 3 | Week 4 |
| --- | --- | --- | --- | --- | --- |
| 1 | Weekly classroom-wise master schedule  [Note: Attach this as a separate sheet and note down any planned changes to this schedule] |  |  |  |  |
| 2 | Any days and timings when students might be free due to unavailability of teacher(s) |  |  |  |  |
| 3 | Any special events planned  [Note the date and timings] |  |  |  |  |
| 3a | Programs for the students |  |  |  |  |
| 3b | Programs for the teachers |  |  |  |  |
| 3c | Parent-teacher meeting |  |  |  |  |
| 3d | Sports event |  |  |  |  |
| 3e | Administrative meetings |  |  |  |  |
| 3f | Programs and performances for students |  |  |  |  |
| 3g | Special classes for students |  |  |  |  |
| 3h | Scheduled field trips, camps, etc. |  |  |  |  |
| 3i | Any other planned events |  |  |  |  |
| 3j | Any unplanned events |  |  |  |  |
| 4 | Examination schedule |  |  |  |  |
| 5 | Scheduled holidays |  |  |  |  |
| 6 | Unscheduled holidays |  |  |  |  |
| 7 | Details on any school-level events/celebrations that might disrupt the classroom schedule |  |  |  |  |
| 8 | Details on any changes to regular school opening and closing timings |  |  |  |  |
